# “A picture is worth a thousand words”: smoking in multi-unit housing in Israel

**DOI:** 10.1186/s13584-023-00574-9

**Published:** 2023-08-07

**Authors:** Mitchell Zeller

**Affiliations:** Retired director, Center for Tobacco Products, U.S. Food and Drug Administration, Silver Spring, MD USA

**Keywords:** Tobacco smoke incursion, Multi-unit housing, Policy change

## Abstract

A brief commentary on the need for policy change by the Israeli government to address the problem of tobacco smoke incursion in multi-unit housing. The commentary also includes a call for enhanced products, programs, and services to help smokers in Israel quit.

There is an old expression that “A picture is worth a thousand words.” No truer words have been spoken or written about the reality of tobacco smoke incursion in Israel than Fig. 1 which accompanies a recent paper by Theitler et al. [1].

**Fig. 1:**
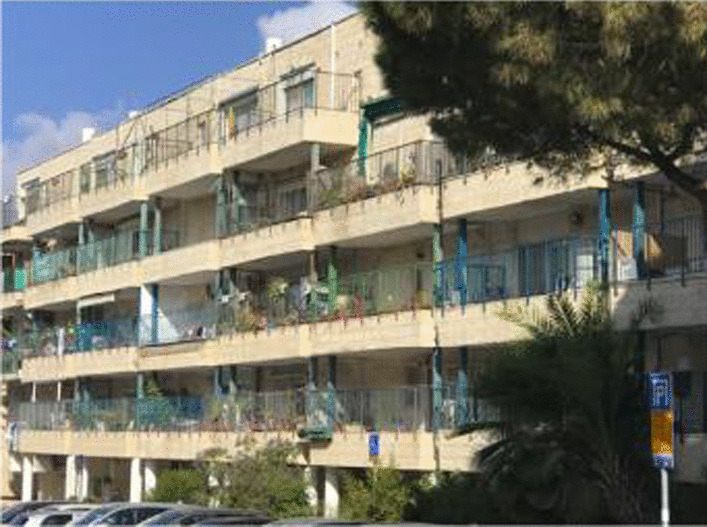
Theitler, Rees, Raz, Bitan, Rosen

There are several important findings from this paper with profound public health policy implications for decision makers in Israel. First, living in multi-unit housing is linked to a 3.6 times greater likelihood of experiencing tobacco smoke incursion than living in a private home. Second, and Fig. 1 provides compelling evidence why, “37.9% [of those acknowledging tobacco smoke incursion] reported that the smoker was located on a floor below, 17.9% reported that the smoker was on the same floor, 15.8% reported that the smoke came from the floor above” [1].

These results take on added significance when one realizes that Israel has one of the world’s highest rates of population density [2], and approximately three-quarters of Israelis live in multi-unit housing [3]. This latter statistic is likely even higher for those who are less well-off economically. Furthermore, it has been estimated that the smoke from one porch in Fig. 1 can be in the air within nine meters of *more than ten* nearby apartments [1]. (emphasis added.)

Exposure to secondhand smoke is no mere nuisance. As described by Theitler, et al., secondhand smoke has been demonstrated to be related to significant morbidity and mortality among nonsmokers exposed to it [1].

I believe that the Israeli government should address this ongoing public health threat. Unfortunately, in important pending litigation before the Israeli Supreme Court, Case Number 1416/21, the Ministry of Health is opposing efforts to provide legal protection for people exposed to tobacco smoke incursion into their homes. The practical impact of the Ministry’s legal posture is to encourage smokers to do the most convenient thing, which is to keep smoking on their porches. All available evidence makes clear that this is not a solution. Worse, it could exacerbate the problem.

I see this as both a public health and a moral imperative and feel that Israeli citizens have the right to breathe clean air in their residences. Particularly vulnerable subpopulations, such as children and the elderly, are especially deserving of these protections. Accordingly, I believe that the Ministry of Health should reconsider its position.

The status quo is troubling. The evidence presented in the paper by Theitler el al makes clear the extent of tobacco smoke incursion among study participants, and the extent to which they were troubled by that exposure or believed that it was harmful to them.

Smoking in areas in multi-unit housing where it reaches neighbors is not a public health solution. Thinking that smoking on apartment porches solves the problem is akin to believing that having a roped-off urinating section of a swimming pool solves the problem of urination in pools. I am not suggesting this is a perfect analogy. Nor am I making light of the current situation regarding secondhand smoke exposure. But it would not work in pools, and porch smoking will not solve the very real and serious health problems caused by involuntary exposure to someone else’s cigarette smoke which penetrates an individual’s residence.

One hopes this unresolved issue would also lead the Israeli government to focus on the underlying problem, namely, ongoing smoking. That is the true root cause, and data show that there has been no decrease in smoking in Israel in over the past decade, unlike in the U.S., for example, where smoking has decreased from 20.9 to 11.5% since 2005 [5]. No matter which way the Israeli government chooses to address the secondhand smoke dilemma, including tobacco smoke incursion in homes and exposure from other sources, it must also reconsider and expand efforts to help smokers quit, and to prevent smoking initiation.

Of equal importance is the need for all sectors of Israeli society to come together, under the Ministry’s leadership, to denormalize smoking anywhere in the multi-unit housing setting in the same way that smoking on airplanes and in cars has been denormalized. Were that to happen, and coupled with enhanced products, programs, and services to help smokers quit, Israel could solve a pressing public health problem.

It should all start with the Ministry supporting legal protection for residents in multi-unit housing exposed to cigarette smoke. Next, the Ministry should proactively take charge of solving the problem. This can be done by the Ministry working to accelerate the denormalization of smoking in, or on the grounds of, multi-unit housing, and anyplace near other individuals, whether indoors or outdoors. In addition, it should strengthen national cessation efforts and actions to prevent youth initiation.

## Conclusion

I believe that Israelis living in multi-unit housing have a fundamental right to breathe clean air in and around their homes. They currently are at risk. There are meaningful actions their government could and should take to ensure this right and improve the current and future health of the country’s population.

## Data Availability

The data are available from the authors upon reasonable request.
